# Health-related quality of life among Indian population: The EQ-5D population norms for India

**DOI:** 10.7189/jogh.13.04018

**Published:** 2023-02-17

**Authors:** Gaurav Jyani, Shankar Prinja, Basant Garg, Manmeet Kaur, Sandeep Grover, Atul Sharma, Aarti Goyal

**Affiliations:** 1 Department of Community Medicine and School of Public Health, Postgraduate Institute of Medical Education and Research (PGIMER), Chandigarh, India; 2 National Health Authority, Ministry of Health and Family Welfare, Government of India, New Delhi, India; 3 Department of Psychiatry, Postgraduate Institute of Medical Education and Research (PGIMER), Chandigarh, India

## Abstract

**Background:**

The EuroQol 5 dimensions (EQ-5D) is the most used generic health-related quality of life (HRQoL) instrument for measuring population health and health outcomes. Since there are no EuroQol 5 dimensions 5 levels (EQ-5D-5L) population norms available for India, this study developed the Indian population norms for the EQ-5D-5L. The potential influencing factors of HRQoL of the Indian population have been identified.

**Methods:**

The data was collected alongside the Indian EQ-5D-5L valuation study (Development of an EQ-5D Value Set for India Using an Extended Design: DEVINE Study). A cross-sectional survey of 3548 adult respondents was conducted across five states of India, in which respondents were asked to report their own health states using the EQ-5D-5L descriptive system and the EuroQol Visual Analog Scale (EQ VAS). The utility score was calculated using the EQ-5D-5L value set based on the preferences of the Indian population. Norm scores were generated for age, sex, and other important socio-demographic variables. The proportion of patients reporting problems in different dimensions of EQ-5D-5L was assessed. The impact of socio-economic determinants on health-related quality of life was evaluated using multiple linear regression.

**Results:**

The mean EQ VAS score of the Indian population is 75.18 (95% confidence interval (CI) = 74.50-75.90), whereas mean utility score is 0.848 (95% CI = 0.840-0.857). The EQ VAS scores, and utility scores decreased with age. Males reported higher EQ VAS values than females. The highest mean utility score was observed for males of <20 years (0.936), whereas the lowest mean score was observed for females of >70 years (0.488). The mean VAS score ranged between 85.24 for females of <20 years and 50.67 for females of >70 years. Highest problems were reported in the dimension of “pain / discomfort”, closely followed by “anxiety / depression”. Age, educational qualification, marital status, substance abuse, presence of ailments, state / region of residence, number of dependent members in the household, and time spent on mobile are the significant determinants of HRQoL of Indian population.

**Conclusions:**

These population norms will be used as reference values for comparative purposes in future Indian studies. Economic evaluations can use these average age-specific HRQoL population norms to value the health-state of not having the specific disease under investigation.

In the contemporary medical and public health practice, health-related quality of life (HRQoL) has emerged amongst the most important measurable outcomes of health programs and interventions in the last couple of decades. It has been seen as a reliable measure to account for actual improvement in patients’ overall health status [1]. Moreover, it has been emphasized that health care policy planning must rely on relevant information about the health state of patient groups as well as the preferences of the general population [2]. In this regard, the EuroQol 5 dimensions (EQ-5D) is the most widely used instrument across the globe for assessment of HRQoL in economic, clinical, and population health studies [3]. The EQ-5D descriptive system comprises five dimensions: mobility, self-care, usual activities, pain / discomfort, and anxiety / depression [4,5]. It has two versions, a three-level EQ-5D (EQ-5D-3L), and a five-level EQ-5D (EQ-5D-5L). Although EQ-5D-3L has been widely used, it is reported to suffer from ceiling effects (i.e. the percentage of respondent reporting the best possible health state on EQ-5D) and measurement insensitivity [6]. By increasing the number of levels in the descriptive system, EQ-5D-5L has demonstrated reduced ceiling effects and improved discriminatory power in comparison to EQ-5D-3L [7-9]. In addition to classifying health states in terms of the 5 dimensions of health, EQ-5D permits the valuation of these health states. This is accomplished from both the respondent’s own perspective by using a Visual Analogue Scale (EQ VAS), and from the perspective of the general public by attaching the appropriate EQ-5D index score / utility score to the described health state of the respondent.

EQ-5D has been used to measure population health in many countries, and population norms have been established by age, gender and socio-economic status [10]. The EQ-5D population norms are reported for EQ VAS and EQ-5D utility scores, and for self-reported problems on each of the five dimensions of the EQ-5D descriptive system, all classified by age and gender [10]. These EQ-5D norms can be used as reference data to compare patients with specific conditions, and to assess the burden of the disease in question. A set of population norm scores provides an important reference point for clinical and health economic research outcomes, as the effects of medical conditions and / or treatments can be quantified by comparing patients and / or intervention groups with the general population [11].

Furthermore, it has been argued that many cost-utility analyses use the upper anchor of the scale (equivalent to perfect health) to denote only the absence of the diseases / health condition being investigated, and erroneously assign the quality of life weight of 1.0 to absence of a particular disease [12,13]. Such an assumption ignores the fact that an individual may still host other chronic and acute conditions, and be comorbid with the condition being analysed. The absence of a particular health condition is not same as the perfect health. Therefore, it has been recommended that analysts undertaking cost-utility analyses without access to primary data from treated patients should use the average age-specific HRQoL weights from population norm studies to represent the state of not having a particular disease [12]. However, presently there are no EQ-5D-5L norms for the Indian population, which not only hampers the effective conduct of cost-utility analyses in the country, but also hinders the use of EQ-5D-5L in India, which is a recommended instrument for assessment of HRQoL during the conduct of health technology assessment (HTA) in the country [14].

The objective of this paper is to provide population norm, including the prevalence of EQ-5D-5L health problems, and EQ-VAS and EQ index scores by age and gender, in the Indian population. These population norms provide estimate of HRQoL of the Indian population, disaggregated by the descriptive statistics in order to provide flexibility to the researchers when using the EQ-5D norms for comparative purposes. These population norms can be used as reference data to compare profiles for patients with specific conditions with data for the average person in the general population in a similar age and / or gender group. In addition to assessing the HRQoL, we also examined the determinants of HRQoL among the Indian population.

## METHODS

### Sampling and recruitment

The data for generating the EQ-5D-5L population norms for India was collected as a part of the Indian EQ-5D-5L valuation study (Development of an EQ-5D Value set for India using the Extended design: DEVINE study) [15,16]. In the DEVINE study, the respondents reported self-reported health status data, health-state valuation data, and socio-demographic data. Whereas the health-state valuation data was used to establish the EQ-5D-5L value set for India [16], self-reported health status data and socio-demographic data was used to establish the population norms for the Indian population. To obtain a sample representative of the country’s population, the sample selection involved a rigorous process wherein the selection was made at five different levels, i.e. at the level of regions, districts, primary sampling units (PSUs), households, and the individuals to be interviewed. The study was undertaken in five regions / states of India. The selection of regions was based on three criteria: income, health status, and geographical representation. The respondents were selected using a multistage stratified random sampling technique. The detailed approaches describing the selection of study settings, sample size, interview process and quality control have been reported separately [15,16].

### Study instruments

The health-related quality of life of the Indian population was measured using EQ-5D, which comprises of the EQ-5D-5L descriptive system and EQ VAS. The EQ-5D-5L descriptive system covers five dimensions: mobility, self-care, usual activity, pain / discomfort, and anxiety / depression, and each dimension has five possible levels of response, i.e. no problems, slight problems, moderate problems, severe problems, and extreme problems [4]. Based on the level of difficulty reported by the individual among the five dimensions, an EQ-5D-5L health state is defined for it, which is represented as a five-digit number (e.g. 11 111, 11 112, etc.), wherein each digit represents the level of problem in the respective dimension. In addition, all the respondents were also asked to rate their health on the day of interview between 0-100 through the EQ VAS [17]. It consists of a 20 cm vertical line with clearly defined endpoints. The scores represent the ordinal rankings of the health outcomes, where “0” denotes the worst health state and “100” denotes the best health state from the patients’ perspective. The EuroQol Group provided English as well as officially translated versions of EQ-5D in four Indian languages (Hindi, Gujarati, Tamil and Odia), which are used in the regions / states where in the data collection was undertaken.

### Data collection

The computer assisted personal interviewing (CAPI) techniques was used for the data collection, wherein each respondent was interviewed face-to-face by a trained interviewer using the EuroQol valuation technology (EQ-VT) [16,18,19]. The study participants were interviewed about: (1) self-reported health using the EQ-5D-5L descriptive system and EQ VAS, (2) questions on age, gender, and prior experience of serious illness, (3) valuation exercise using 10 composite time trade-off (c-TTO) and seven discrete choice (DCE) experiments, (4) questions on socio-economic and demographic characteristics, pre-existing medical history, habits and beliefs, and other attributes of the respondents. During the process of interviews, interviewers also carried along a graphical illustration (Likert scale smileys) explaining the five levels of severity. This was done keeping in mind the literacy status of participants to read the description of the health states on the screen. They could look at the graphical illustration supported by the description provided by the interviewer and make an informed decision. The data collected in the first and fourth part of the interview was used in this paper. To ensure the quality and uniformity of the data collection process, the study followed intensive training and implemented stringent quality control (QC) measures. The recommendations of the latest EQ-VT protocol were followed to standardize the data collection process across different regions of the country [19-21].

### Data analysis

The analysis was done using STATA-13 statistical package. For each respondent, the EQ-5D-5L health state and the EQ VAS score were directly observed from the respondent’s self-reported questionnaire. The proportion of respondents reporting problems in different attributes of EQ-5D-5L was assessed. The EQ-5D-5L health state of the respondent was used to produce a single EQ index score / quality of life score / utility score between <0 and 1. Utility score of 1 means perfect health and 0 implies death. The Indian EQ-5D-5L value-set was used to estimate the utility score for the distinct health states [16]. In the EQ-5D-5L value-set, the utility score of all the possible 3125 health states have been provided. For each respondent, we derived their corresponding utility scores from their self-reported health states.

The descriptive statistics of EQ-5D-5L health state, EQ VAS score, and utility score were calculated for the whole sample, and by different socio-demographic variables (age, gender, employment status, etc.). For comparison, we categorized age into age groups following other countries’ population norms studies [22-25]. Descriptive analyses were conducted to assess data characteristics of the respondents. The normality of the parameters was tested using the Kolmogorov-Smirnov test. As the variables were normally distributed, so we used one way analysis of variance (ANOVA) to assess the statistical significance among the mean HRQoL of respondents of different age, religion, state (province), marital status, employment status, educational status, number of dependent and earning members in household, and with presence of ailments or substance abuse, whereas the Student’s *t* test was used was used to see the difference in mean HRQoL among the patients of different residences (rural / urban), wherein the level of significance was set at 5%. For each socio-demographic variable, the percentage of reported problem in EQ-5D dimension, the means (m) (and 95% confidence interval (CI)) of EQ VAS score and utility scores were calculated to generate EQ-5D-5L population norms for the Indian population.

To assess the determinants of health- related quality of life (utility score), multiple linear regression model using ordinary least square method was used. As the assumptions of normality of the error term, and presence of homoscedasticity were violated, thereby, to avoid biased and inconsistent estimates, we used generalized linear model method using maximum likelihood estimation approach for estimation of associations [26]. This approach relaxes the assumptions of normality of response and residuals, and provides the consistent estimators for further use. The relationship in the generalized linear model is assumed to be

*Y = g (b_0_ + b_1_X_1_ + b_2_X_2_ +∙∙∙ b_k_X_k_) + e *(1)

where *X_i_* is the value of the *i*^th^ predictor, *e* is the error, and g () is a function. Formally, the inverse function of *g* (), say *f* (), is called the link function; so that

*f* (*μ_y_*) = *b_0_ + b_1_X_1_ + b_2_X_2_ +∙∙∙ b_K_X_k _*(2)

where *μ_y_* stands for the expected value of *y*. Based on the distribution of the τvariable, we used identity link function for the current analysis [27,28]. We used utility score as a dependent variable, while remaining variables of state, age, income, number of dependent members, total time spent on laptop, total time spent on mobile, gender, educational qualification, marital status, religion, occupation, substance abuse, and presence of ailments were used as predictors to understand their influence on the response variable.

### Ethical considerations

The study was performed in accordance with the ethical standards as laid down in the 1964 Declaration of Helsinki. All interviews were conducted with care and sensitivity and with respect for participants’ ethnicity, religion, language, sexual orientation or literacy level. Participants were first presented the study’s participant information sheets and given enough time to read or be read the participant information sheet and to ask questions and discuss concerns regarding potential participation in the study. Thereafter, the signatures of the participants were obtained on the informed consent forms, and the interviews were conducted after it, all within one visit. The ethical approval to conduct the study was obtained from the Institutional Ethics Committee of Postgraduate Institute of Medical Education and Research, Chandigarh, India, vide reference No. PGI/IEC/2018/001629.

## RESULTS

### Sample characteristics

A total of 3548 interviews were conducted between June 2019 and March 2020 using the latest available EQ-VT v2.1 system [18,19,29]. After removing the incomplete / practice / pilot interviews and interviews flagged due to respondents’ lack of understanding, 2311 interviews were considered in the final analysis. The interviews which were not included in the final analysis were predominantly pilot interviews (n = 788). Such a large pilot was conducted to ensure protocol compliance and minimize the interviewers’ effect, considering the limited literacy rate of the Indian population, and to standardize the data collection process across all the study sites as well as interviewers. The remaining interviews which were not included in the analysis were either incomplete (n = 98) or were requested for non-inclusion by the respondents because of their lack of understanding (n = 301), or were flagged by the interviewers due to the respondents’ lack of involvement (n = 50). The mean age of the respondents was 42 years (standard deviation (SD) = 16 years), the age ranged between 18 years to 82 years old. Females comprised 51.1% of the sample. Majority of the respondents were married (70.6%) and resided in rural areas (68.7%). The detailed socio-demographic information of the respondents is presented in **Table 1**.

**Table 1:** Socio-demographic characteristics of the respondents

Characteristics		Number	Percentage
**Age group (years)**	<20	97	4.2%
	20-29	625	27.0%
	30-39	518	22.4%
	40-49	467	20.2%
	50-59	326	14.1%
	60-69	188	8.1%
	>70	90	3.9%
**Gender**	Male	1129	48.9%
	Female	1178	51.1%
**Educational status**	Illiterate	250	10.8%
	Primary	296	12.8%
	Middle	395	17.1%
	Matric	438	19.0%
	Senior secondary	405	17.5%
	Graduate and above	527	22.8%
**Marital status**	Married	1631	70.6%
	Never married	498	21.5%
	Widowed / divorced	182	7.9%
**Dependent members in household**	1	218	9.4%
	2-3	1061	45.9%
	4-5	762	33.0%
	More than 5	270	11.7%
**Area of residence**	Urban	724	31.3%
	Rural	1587	68.7%
**Employment status**	Non-employed	1176	50.9%
	Self-employed	734	31.8%
	Employed in public sector	178	7.7%
	Employed in private sector	223	9.6%
**Substance abuse**	Alcohol	115	5.0%
	Tobacco (smoking / smokeless)	623	27.0%
	Both alcohol and tobacco	33	1.4%
	None	1540	66.6%
**Presence of ailments**	No ailment	1362	58.9%
	Chronic ailment	371	16.1%
	Acute ailment	320	13.8%
	Both acute and chronic	258	11.2%
**Religion**	Hindu	2046	88.5%
	Muslim	119	5.1%
	Christian	115	5.0%
	Other	31	1.3%
**Earning members in household**	Single earning member	1172	50.7%
	Multiple earning members	1139	49.3%
**Region / state**	Haryana	432	18.7%
	Gujarat	394	17.0%
	Odisha	509	22.0%
	Tamil Nadu	460	19.9%
	Uttar Pradesh	516	22.3%
**Total**		2311	100.0%

### Health related quality of life among Indian population

The analysis of the health profiles revealed that maximum problems were reported in the dimensions of pain / discomfort and anxiety / depression. The percentages of respondents reporting “no problems” were 67.36% for mobility, 85.57% for self-care, 70.09% for usual activity, 45.3% for pain / discomfort, and 45.38% for anxiety / depression (**Table 2**). The mean EQ VAS and utility scores among the respondents were 75.18 (SD = 16.42) and 0.849 (SD = 0.212), respectively.

**Table 2:** Respondents reporting problems in different attributes of EuroQol 5 dimensions (EQ-5D) descriptive system

	Mobility			Self-care			Usual activity			Pain / discomfort			Anxiety / depression			
	Male	Female	Overall	Male	Female	Overall	Male	Female	Overall	Male	Female	Overall	Male	Female	Overall
**No problems**	69.71%	65.11%	67.36%	86.09%	85.06%	85.57%	71.48%	68.76%	70.09%	48.63%	42.11%	45.30%	47.12%	43.72%	45.38%
**Slight problems**	19.31%	21.73%	20.55%	10.45%	12.14%	11.31%	19.13%	20.46%	19.81%	31.00%	31.32%	31.17%	24.80%	28.44%	26.66%
**Moderate problems**	8.24%	10.10%	9.19%	2.57%	1.87%	2.21%	7.35%	8.32%	7.85%	17.18%	21.05%	19.16%	20.19%	18.68%	19.42%
**Severe problems**	2.21%	2.63%	2.43%	0.80%	0.76%	0.78%	1.95%	2.38%	2.17%	3.10%	5.18%	4.16%	6.91%	8.15%	7.54%
**Extreme problems**	0.53%	0.42%	0.48%	0.09%	0.17%	0.13%	0.09%	0.08%	0.09%	0.09%	0.34%	0.22%	0.97%	1.02%	1.00%

**Table 3** and **Table 4** show the percentage of reported problems for each severity level and EQ-5D dimension, and the mean (SD) of EQ VAS and utility scores for males and females by age groups, respectively. In both male and female groups, the number of problems increased with age in the dimensions of mobility, self-care, usual activities, and pain / discomfort. In contrast, anxiety / depression was more prevalent in younger age groups in males. In females, the problems of anxiety / depression increased with age. As could be expected, the mean EQ VAS scores and utility scores decreased with age. Males reported higher EQ-VAS values than males. The highest mean utility score was observed for males of <20 years (0.936), whereas the lowest mean score was observed for females of >70 years (0.488). The mean VAS score ranged between 85.24 for females of <20 years and 50.67 for females of >70 years.

**Table 3:** Percentage of population reporting problems of levels 1 to 5 across different dimensions, EuroQol Visual Analogue Scale (EQ VAS) & utility score by age groups for males

EQ-5D Dimension		Age groups							Total n = 1129
		**<20, n = 48**	**20-29‚ n = 308**	**30-39‚ n = 230**	**40-49‚ n = 226**	**50-59‚ n = 162**	**60-69‚ n = 101**	**>70‚ n = 54**	
**Mobility**	No problems	89.6%	89.3%	78.3%	69.0%	50.6%	36.6%	25.9%	69.7%
	Slight problems	6.3%	6.8%	15.2%	23.0%	34.6%	38.6%	22.2%	19.3%
	Moderate problems	4.2%	2.9%	4.3%	4.9%	13.0%	19.8%	37.0%	8.2%
	Severe problems	0.0%	0.6%	2.2%	1.3%	1.9%	5.0%	13.0%	2.2%
	Unable to	0.0%	0.3%	0.0%	1.8%	0.0%	0.0%	1.9%	0.5%
	χ^2^ (*P*-value)	275.65 (<0.001)							
**Self-care**	No problems	100.0%	93.5%	91.3%	86.7%	77.8%	74.3%	53.7%	86.1%
	Slight problems	0.0%	4.9%	7.8%	10.2%	17.3%	14.9%	35.2%	10.5%
	Moderate problems	0.0%	1.0%	0.9%	1.3%	3.7%	9.9%	9.3%	2.6%
	Severe problems	0.0%	0.6%	0.0%	1.8%	1.2%	1.0%	0.0%	0.8%
	Unable to	0.0%	0.0%	0.0%	0.0%	0.0%	0.0%	1.9%	0.1%
	χ^2^ (*P*-value)	135.09 (<0.001)							
**Usual activity**	No problems	87.5%	84.7%	73.5%	66.8%	67.9%	52.5%	38.9%	71.5%
	Slight problems	10.4%	10.4%	20.4%	25.2%	18.5%	32.7%	22.2%	19.1%
	Moderate problems	2.1%	4.5%	5.2%	6.2%	11.1%	11.9%	22.2%	7.4%
	Severe problems	0.0%	0.3%	0.9%	1.8%	2.5%	3.0%	14.8%	1.9%
	Unable to	0.0%	0.0%	0.0%	0.0%	0.0%	0.0%	1.9%	0.1%
	χ^2^ (*P*-value)	154.50 (<0.001)							
**Pain / discomfort**	No problems	70.8%	70.8%	49.6%	38.1%	34.6%	29.7%	20.4%	48.6%
	Slight problems	18.8%	19.8%	36.5%	38.9%	35.2%	34.7%	29.6%	31.0%
	Moderate problems	8.3%	7.1%	12.2%	19.0%	27.8%	31.7%	37.0%	17.2%
	Severe problems	2.1%	2.3%	1.7%	4.0%	2.5%	4.0%	11.1%	3.1%
	Extreme problems	0.0%	0.0%	0.0%	0.0%	0.0%	0.0%	1.9%	0.1%
	χ^2^ (*P*-value)	180.79 (<0.001)							
**Anxiety / depression**	No problems	79.2%	56.5%	40.4%	38.5%	35.8%	50.5%	57.4%	47.1%
	Slight problems	6.3%	23.1%	31.3%	25.7%	27.2%	22.8%	16.7%	24.8%
	Moderate problems	6.3%	14.9%	20.9%	25.7%	29.0%	18.8%	13.0%	20.2%
	Severe problems	8.3%	4.9%	7.4%	8.4%	7.4%	6.9%	7.4%	6.9%
	Extreme problems	0.0%	0.6%	0.0%	1.8%	0.6%	1.0%	5.6%	1.0%
	χ^2^ (*P*-value)	80.03 (<0.001)							
**EQ VAS**	Mean	84.44	83.84	79.77	73.61	69.89	65.47	56.54	76.04
	95% CI	(80.46-88.42)	(82.44-85.25)	(78.12-81.41)	(71.85-75.38)	(67.67-72.10)	(61.98-68.95)	(51.26-61.81)	(75.11-76.96)
	F-test (*P*-value)	55.348 (<0.001)							
**Utility score**	Mean	0.936	0.920	0.882	0.833	0.814	0.780	0.643	0.855
	95% CI	(0.899-0.973)	(0.904-0.936)	(0.863-0.901)	(0.805-0.862)	(0.785-0.843)	(0.739-0.820)	(0.550-0.736)	(0.843-0.866)
	F-test (*P*-value)	25.068 (<0.001)							

EQ-5D – EuroQol 5 dimensions, CI – confidence interval, EQ VAS – EuroQol Visual Analogue Scale

**Table 4:** Percentage of population reporting problems of levels 1 to 5 across different dimensions, EuroQol Visual Analogue Scale (EQ VAS) & utility score by age groups for females

EQ-5D Dimension		Age groups							Total n = 1178
		**<20, n = 49**	**20-29‚ n = 316**	**30-39‚ n = 288**	**40-49‚ n = 241**	**50-59‚ n = 164**	**60-69‚ n = 87**	**>70‚ n = 33**
**Mobility**	No problems	87.8%	86.4%	75.7%	63.1%	38.4%	17.2%	9.1%	65.1%
	Slight problems	8.2%	11.4%	13.5%	23.7%	41.5%	46.0%	36.4%	21.7%
	Moderate problems	4.1%	1.9%	8.7%	10.0%	16.5%	28.7%	30.3%	10.1%
	Severe problems	0.0%	0.3%	1.7%	3.3%	3.7%	6.9%	15.2%	2.6%
	Unable to	0.0%	0.0%	0.3%	0.0%	0.0%	1.1%	9.1%	0.4%
	χ^2^ (*P*-value)	351.92 (<0.001)							
**Self-care**	No problems	95.9%	94.3%	90.3%	89.6%	73.8%	58.6%	27.3%	85.1%
	Slight problems	4.1%	5.1%	8.3%	9.1%	23.2%	32.2%	39.4%	12.1%
	Moderate problems	0.0%	0.6%	0.7%	0.8%	2.4%	5.7%	21.2%	1.9%
	Severe problems	0.0%	0.0%	0.7%	0.4%	0.6%	3.4%	6.1%	0.8%
	Unable to	0.0%	0.0%	0.0%	0.0%	0.0%	0.0%	6.1%	0.2%
	χ^2^ (*P*-value)	287.63 (<0.001)							
**Usual activity**	No problems	95.9%	78.8%	75.7%	68.5%	57.9%	34.5%	18.2%	68.8%
	Slight problems	4.1%	17.7%	17.7%	21.2%	23.8%	41.4%	18.2%	20.5%
	Moderate problems	0.0%	3.2%	5.6%	8.3%	14.0%	16.1%	45.5%	8.3%
	Severe problems	0.0%	0.3%	1.0%	2.1%	4.3%	8.0%	15.2%	2.4%
	Unable to	0.0%	0.0%	0.0%	0.0%	0.0%	0.0%	3.0%	0.1%
	χ^2^ (*P*-value)	235.03 (<0.001)							
**Pain / discomfort**	No problems	63.3%	58.2%	45.8%	37.8%	26.8%	12.6%	9.1%	42.1%
	Slight problems	22.4%	26.3%	34.4%	34.0%	34.8%	29.9%	33.3%	31.3%
	Moderate problems	12.2%	13.6%	14.2%	23.7%	30.5%	46.0%	33.3%	21.1%
	Severe problems	0.0%	1.6%	5.2%	4.1%	7.9%	11.5%	24.2%	5.2%
	Extreme problems	2.0%	0.3%	0.3%	0.4%	0.0%	0.0%	0.0%	0.3%
	χ^2^ (*P*-value)	168.86 (<0.001)							
**Anxiety / depression**	No problems	71.4%	52.2%	40.3%	39.4%	37.8%	35.6%	33.3%	43.7%
	Slight problems	10.2%	25.6%	33.0%	27.8%	29.3%	31.0%	36.4%	28.4%
	Moderate problems	10.2%	16.8%	17.4%	23.7%	20.1%	20.7%	12.1%	18.7%
	Severe problems	6.1%	4.4%	8.3%	8.3%	11.6%	11.5%	18.2%	8.1%
	Extreme problems	2.0%	0.9%	1.0%	0.8%	1.2%	1.1%	0.0%	1.0%
	χ^2^ (*P*-value)	50.14 (<0.001)							
**EQ VAS**	Mean	85.24	81.52	77.23	74.02	66.00	60.64	50.67	74.52
	95% CI	(81.54-88.95)	(80.00-83.04)	(75.65-78.80)	(72.03-76.01)	(63.58-68.42)	(57.06-64.22)	(45.54-55.8)	(73.58-75.47)
	F-test (*P*-value)	55.081 (<0.001)							
**Utility score**	Mean	0.922	0.908	0.858	0.831	0.766	0.669	0.488	0.831
	95% CI	(0.885-0.959)	(0.894-0.921)	(0.837-0.879)	(0.807-0.855)	(0.731-0.801)	(0.611-0.728)	(0.352-0.623)	(0.819-0.843)
	F-test (*P*-value)	42.292 (<0.001)							

EQ-5D – EuroQol 5 dimensions, CI – confidence interval, EQ VAS – EuroQol Visual Analogue Scale

Beside age and gender, **Table 5** shows the mean (SD) of EQ VAS and utility scores by other socio-demographic characteristics. People with lower educational qualification also had lower HRQoL, as both the utility score and EQ VAS score showed an increasing trend with an increase in the educational qualification. People who were never married had better HRQoL (both the EQ VAS score and utility score) as compared to people who are married, followed by widowed / divorced. No difference between the HRQoL of the people living in rural and urban areas was observed. Analysis of HRQoL among different employment groups revealed that people employed in private sector had the best HRQoL, whereas unemployed people reported the worst HRQoL. As far as presence of ailments is concerned, as could be expected, people with no ailment reported to have the best HRQoL, followed by people with acute ailments, people with chronic ailments, and people with both acute and chronic ailments, respectively. The detailed analysis of HRQoL (utility score and EQ VAS score) among Indian population across different socio-demographic groups has been presented in **Table 5**.

**Table 5:** Health-related quality of life (utility score and EuroQol Visual Analogue Scale (EQ VAS) score) among Indian population across different socio-demographic groups

Characteristics		Utility score		EQ VAS score	
		**Mean ± SD**	*P*-value*	**Mean ± SD**	*P*-value*
**Educational status**	Illiterate	0.708 ± 0.301	<0.001	62.62 ± 18.25	<0.001
	Primary	0.816 ± 0.233		70.39 ± 16.42	
	Middle	0.835 ± 0.201		73.33 ± 15.61	
	Matric	0.854 ± 0.199		75.66 ± 15.75	
	Senior secondary	0.897 ± 0.141		78.78 ± 13.82	
	Graduate and above	0.902 ± 0.177		82.03 ± 13.85	
**Marital status**	Married	0.848 ± 0.188	<0.001	74.22 ± 15.47	<0.001
	Never married	0.917 ± 0.150		83.38 ± 13.42	
	Widow / divorce	0.665 ± 0.383		61.27 ± 20.06	
**Dependent members in household**	1	0.814 ± 0.318	<0.001	73.87 ± 18.99	0.271
	2-3	0.861 ± 0.186		75.82 ± 15.76	
	4-5	0.855 ± 0.193		74.62 ± 16.21	
	More than 5	0.809 ± 0.246		75.27 ± 17.24	
**Area of residence**	Urban	0.844 ± 0.244	0.564	75.74 ± 17.14	0.276
	Rural	0.850 ± 0.196		74.92 ± 16.07	
**Employment status**	Unemployed	0.833 ± 0.240	<0.001	74.06 ± 17.65	<0.001
	Self-employed	0.852 ± 0.183		74.87 ± 15.15	
	Employed in public sector	0.884 ± 0.166		78.07 ± 14.63	
	Employed in private sector	0.890 ± 0.165		79.72 ± 13.83	
**Substance abuse**	Alcohol	0.907 ± 0.151	<0.001	80.24 ± 14.92	<0.001
	Tobacco (smoking / smokeless)	0.826 ± 0.203		72.74 ± 15.98	
	Both alcohol and tobacco	0.833 ± 0.337		68.03 ± 21.26	
	None	0.854 ± 0.216		75.94 ± 16.42	
**Presence of ailments**	No ailment	0.902 ± 0.172	<0.001	79.44 ± 14.70	<0.001
	Chronic ailment	0.778 ± 0.242		68.19 ± 17.40	
	Acute ailment	0.819 ± 0.221		75.62 ± 14.89	
	Both acute and chronic	0.704 ± 0.243		62.14 ± 15.13	
**Religion**	Hindu	0.854 ± 0.197	<0.001	75.35 ± 16.08	<0.001
	Muslim	0.834 ± 0.205		77.14 ± 15.63	
	Christian	0.811 ± 0.238		72.95 ± 16.87	
	Other	0.661 ± 0.632		64.48 ± 30.45	
**Earning members in household**	Single earning member	0.856 ± 0.199	0.097	75.72 ± 15.92	0.107
	Multiple earning members	0.841 ± 0.225		74.62 ± 16.90	
**Region / state**	Haryana	0.894 ± 0.183	<0.001	73.94 ± 17.54	<0.001
	Gujarat	0.906 ± 0.165		79.78 ± 15.63	
	Odisha	0.848 ± 0.230		71.54 ± 15.48	
	Tamil Nadu	0.804 ± 0.248		75.02 ± 18.02	
	Uttar Pradesh	0.807 ± 0.196		76.41 ± 14.39	
**Total**		0.849 ± 0.212		75.18 ± 16.42	

EQ VAS – EuroQol Visual Analogue Scale, SD – standard deviation

*P-value is significant when ≤0.05.

### Determinants of health-related quality of life

Multivariable regression analysis showed that the independent variables that predicted the HRQoL / utility values of the respondents were age, educational qualification, marital status (married vs never married), substance abuse (no vs alcohol consumption), presence of ailments, state / region of residence, number of dependent members in the household, and time spent on mobile (**Table 6**). Increased age of the respondents was found to be negatively influencing their HRQoL (Beta = -0.004, *P* < 0.001). Similarly, HRQoL worsens if number of dependent members in the household increases (Beta = -0.007, *P* < 0.001). Similar association was observed between HRQoL, and time spent on mobile. It has also been observed that presence of ailments also negatively influences the HRQoL of an individual. In contrast to it, the improved level of educational attainment was associated with improved HRQoL. The detailed analysis of the association between various socio-demographic variables and HRQoL has been presented in **Table 6**.

**Table 6:** Determinants of health-related quality of life among Indian population

Parameter		Beta	Standard error	95% Wald confidence interval		*P*-value*
				**Lower**	**Upper**	
**Intercept**		1.102	0.0291	1.045	1.159	<0.001
**Age**		-0.004	0.0004	-0.005	-0.003	<0.001
**Income**		-0.001	0.0018	-0.004	0.003	0.732
**Number of dependent members in household**		-0.007	0.0020	-0.011	-0.003	<0.001
**Time spent on laptop**		0.004	0.0029	-0.002	0.009	0.199
**Time spent on mobile**		-0.006	0.0021	-0.010	-0.001	<0.050
**Gender (reference: male)**	Female	-0.010	0.0095	-0.028	0.009	0.302
**Educational qualification (Reference: illiterate)**	Primary	0.066	0.0176	0.032	0.101	<0.001
	Middle	0.065	0.0163	0.033	0.097	<0.001
	Matric	0.045	0.0155	0.014	0.075	<0.050
	Senior secondary	0.050	0.0151	0.021	0.080	<0.001
	Graduate and above	0.040	0.0157	0.009	0.070	<0.050
**Marital status (Reference: married)**	Never married	-0.069	0.0150	-0.098	-0.039	<0.001
	Widow / divorce	-0.019	0.0117	-0.042	0.004	0.114
**Employment (Reference: non-employed)**	Self-employed	0.010	0.0142	-0.018	0.038	0.469
	Public sector	0.020	0.0149	-0.009	0.049	0.178
	Private sector	0.011	0.0097	-0.008	0.031	0.240
**Substance abuse (Reference: none)**	Alcohol	0.073	0.0323	0.010	0.136	<0.050
	Tobacco	0.010	0.0100	-0.009	0.030	0.309
	Both alcohol and tobacco	0.033	0.0180	-0.002	0.068	0.066
**Presence of ailments (Reference: no ailment)**	Chronic ailment	-0.109	0.0137	-0.136	-0.082	<0.001
	Acute ailment	-0.060	0.0116	-0.083	-0.037	<0.001
	Both acute and chronic ailments	-0.064	0.0112	-0.086	-0.042	<0.001
**Religion (Reference: Hindu)**	Muslim	-0.034	0.0338	-0.100	0.032	0.317
	Christian	-0.011	0.0174	-0.045	0.023	0.535
	Other	0.016	0.0170	-0.018	0.049	0.356
**State / region (Reference: Haryana)**	Gujarat	-0.096	0.0131	-0.122	-0.071	<0.001
	Odisha	-0.134	0.0135	-0.161	-0.108	<0.001
	Tamil Nadu	-0.055	0.0124	-0.079	-0.031	<0.001
	Uttar Pradesh	-0.061	0.0132	-0.087	-0.035	<0.001

*P-value is significant when ≤0.05.

## DISCUSSION

This is the first EQ-5D-5L norms study from India. These general population-based norms provide insights into HRQoL status of the Indian population and how HRQoL varies between different socio-economic groups. Consequently, these population norms facilitate the interpretation of the health outcome assessment studies and cost-effectiveness studies which use QALY as a health outcome (cost-utility analyses). As HRQoL instruments measure postulated constructs, these set of norms provide a reference point to interpret an HRQoL study’s results by comparing HRQoL between the general population and patients with specific conditions from similar age and gender groups [30].

More importantly, the present study provides the average age- and gender-specific HRQoL weights as Indian population norms to accurately assign value to the state of not having a particular disease during the conduct of cost-utility analyses. Earlier studies have reported that the incremental cost-effectiveness ratios obtained from the analyses which don’t value the state of not having a particular disease as per the population norms are generally lower, and must be inflated by about 15% to yield to obtain the correct cost / quality adjusted life year (QALY) [12]. It has also been observed that the non-availability of such population norms had mandated the economic evaluations conducted in the past in India to assign the quality of life weight of 1.0 to the health states corresponding to absence of a particular disease [31-33]. The present study aspires to fulfil this longstanding evidence gap hampering the generation of credible health technology assessment (HTA) evidence for transparent health policy making [34].

The population norms for India have demonstrated similarities with the HRQoL population norms of other countries. For instance, it has been observed that the Indian population has reported less problems in the first three dimensions (mobility, self-care, and usual activities) of EQ-5D as compared to the last two dimensions, with pain / discomfort being the most prevalent dimension. Similar trends have been observed in the EQ-5D population norms for China, South Korea, England, Italy, Australia, and Germany [7,24,25,35-37]. Likewise, as observed in other countries, the EQ VAS and utility score declined with increasing age in the Indian population also. In the same way, Indian women reported lower utility score than men, which is a universal finding across almost all the population norms studies [10]. In contrast to these similarities, one observable difference in the findings of the Indian norms study was that in some of the countries, the percentage of reported problems in anxiety / depression uniformly increased with age across both genders [7,35,36,38], whereas in India, among the males, higher levels of anxiety / depression were observed among the younger and middle age groups. One possible explanation for this finding is that the younger generation perceived more psychological pressures than the older generation due to the fast-paced lifestyle. It is worthwhile to mention here that similar findings had also been observed in China [25].

The findings of our study are also in line with the other studies conducted to assess HRQoL of the Indian population. A large-scale household survey conducted in India to assess the self-reported health status has observed that the HRQoL among the Indians decreases with increase in age [39]. A similar trend has been observed in our analysis. This study has also observed that HRQoL of males is better in comparison to females in India, which is in line with our observations. Estimates of HRQoL among different education and religion categories as reported in our study also corresponds with the findings of this survey [39]. Likewise, another study aimed to assess HRQoL among the residents of three large metropolitan cities in India and Pakistan with a large sample size (16 284 adults aged ≥20 years) using EQ VAS had reported the mean EQ VAS score as 74.0 (95% CI = 73.7 to 74.2), which is close to the findings of our nationally representative study (mean EQ VAS = 75.18 (95% CI = 74.50-75.90)) [40]. Likewise, as observed in our study, this study also found lower HRQoL in elderly population as compared to young, in women as compared to men, in unemployed as compared to employed, and in less educated as compared to more educated [40]. Moreover, individuals with chronic conditions reported worse HRQoL than those without any chronic conditions, which is also in line with our findings [40].

The findings of this study offer important guidance for upstreaming the standards of clinical care as well as for shaping the public health policies in the country. Our study has identified pain and mental health (anxiety / depression) as the most important domains inflicting the HRQoL of the Indian population. Although the percentage of population reporting problems in these two dimensions are almost similar, the analysis revealed that the respondents having problems of anxiety / depression are concentrated on the more severe part of the spectrum, highlighting it as the most troublesome aspect of the health (**Table 2**). Our findings support the recent impetus of the government of India towards improvement of mental health of the population, where it has strengthened the allocation of resources by earmarking a budget for mental health and initiated the National Tele-Mental Health Program [41,42].

The entire reasoning around expansion of the coverage of primary health care is currently focused on diseases. The packages of care which are being devised are also following the disease-specific approach. However, there is a whole range of syndromic manifestations of these diseases, which eventually affects the HRQoL of the patients suffering from these diseases. Therefore, in addition to diseases, public health professionals should also pay attention to address the felt needs of a patient. This can be done by understanding that how do the diseases eventually impact the HRQoL of the patients, and which dimensions of health are primarily impacted. This information is critical because this is what counts most from a patient's point of view. Consequently, in the capacity building of the grassroot and field workers, in addition to imparting knowledge on the technique of measurement and treatment of diseases, aspects related to alleviation of symptoms affecting different dimensions of health should also be made an integral component of the curriculum of capacity building.

Likewise, the information on HRQoL of the patients should also be used to assess the quality of care provided by the health care providers. It will inculcate and enhance the merit of value based care in the health-system, which centres on measurement of health outcomes generated in the patients, which can be ascertained by improvement in the HRQoL [43]. Under the Ayushman Bharat Pradhan Mantri Jan Arogya Yojana (AB-PMJAY), which is the largest publicly funded health insurance scheme of the world, the government of India has created a policy which aim to assess the performance of the health care providers on the basis of improvement in the HRQoL of the patients which are treated in these facilities [44]. This policy also envisages that the payment of incentives to these health care providers should also be linked to the improvement in health outcomes of the treated patients, and HRQoL has been considered as an important parameter for the measurement of health outcomes in these patients.

In the same way, as the contemporary clinical practise is also value-based, the outcomes that matter to patients are the true metrics of the quality of care. When these results are tracked and reported, it encourages advancement and the adoption of best practises, thereby raising the bar for clinical care standards. Efforts to improve HRQoL of the patients should focus primarily upon ameliorating pain and anxiety, it has been demonstrated in our study that pain / discomfort and anxiety / depression have the most significant bearing on the HRQoL of the Indian population. The clinical interventions should focus on the control of pain and the relief of anxiety to achieve better patient-centred outcomes.

The rigorous sampling and stringent quality control methods used in the study have led to the sample which is broadly representative of the Indian population [15,16]. This is a predominant strength of the study and is in contrast with most of the previous country level EQ-5D-5L studies which pursued purposive or quota sampling [45-49]. Employing such a rigorous sampling approach has averted the potential issues related to purposive or quota sampling, such as: (1) the chances of missing out respondents who clearly do not fall into any of the quota groups, and (2) the non-random selection of respondents. Moreover, an iterative QC approach was used to obtain high-quality data. At the same time, one of the limitations of our study is that this is a cross-sectional study, which provided insights into relationship between HRQoL data and socio-demographic variables. However, in terms of understanding the causal relationship between variables and controlling for unobserved heterogeneity, longitudinal data are required. Another limitation of our study is that although we have explored the impact of major socio-demographic factors on the HRQoL, yet there can be additional factors determining the HRQoL of the Indian population and which can be explored for their impact on the HRQoL. For instance, caste can be one potential determinant of the HRQoL among Indians. Although in studies conducted on a smaller scale found no significant association between the caste and HRQoL, however this association is yet to be explored in larger samples [50]. Additionally, the assessment of anxiety / depression among the respondents was done on the basis of the question contained in the EQ-5D. However, this is difficult to assess from the survey alone, as it requires a fair anamnesis and examination to assess this aspect properly. Although the patient reported outcome measures (PROMs) cannot replace the clinical diagnosis, yet this study offers very important insights into the status of mental health among the Indian population as it has been established that a single question on anxiety / depressed mood can detect 85%-90% of patients with mental health problems [51,52]. Consequently, the EQ-5D has been adopted for clinical use in certain health systems for screening of mental health as a standardized approach [53]. Nevertheless, since these are single questions in the generic HRQoL instruments that are representative of the dimensions of health, it is an area of prospective research to assess the level of concordance between the generic and disease specific questionnaires in the patients of a particular disease. Lastly, further research is required to assess the impact of using population norms estimates while valuing the health states not having a particular disease on the outcomes of economic evaluations conduced in India.

## CONCLUSIONS

This study has presented the first EQ-5D-5L population norms for India. These population norms provide an important reference point for clinical and health economic research outcomes, as the effects of medical conditions and treatments can be quantified by comparing patients and intervention groups with the general population norms developed as a part of this study. The study also demonstrates the disparities which exist in self-reported health status measured by EQ-5D-5L across different socio-economic groups.
